# Sugarcane mosaic virus orchestrates the lactate fermentation pathway to support its successful infection

**DOI:** 10.3389/fpls.2022.1099362

**Published:** 2023-01-09

**Authors:** Tong Jiang, Kaitong Du, Pei Wang, Xinhai Wang, Lianyi Zang, Dezhi Peng, Xi Chen, Geng Sun, Hao Zhang, Zaifeng Fan, Zhiyan Cao, Tao Zhou

**Affiliations:** ^1^ State Key Laboratory for Agro-Biotechnology, and Ministry of Agriculture and Rural Affairs, Key Laboratory for Pest Monitoring and Green Management, Department of Plant Pathology, China Agricultural University, Beijing, China; ^2^ Collaborative Innovation Center of Fruit and Vegetable Quality and Efficient Production in Shandong, Shandong Agricultural University, Tai’an, China; ^3^ State Key Laboratory of North China Crop Improvement and Regulation, Hebei Agricultural University, Baoding, Hebei, China

**Keywords:** metabolomics, sugarcane mosaic virus, maize, anaerobic glycolysis, lactate dehydrogenase, viral replicase complexes, lactate

## Abstract

Viruses often establish their own infection by altering host metabolism. How viruses co-opt plant metabolism to support their successful infection remains an open question. Here, we used untargeted metabolomics to reveal that lactate accumulates immediately before and after robust sugarcane mosaic virus (SCMV) infection. Induction of lactate-involved anaerobic glycolysis is beneficial to SCMV infection. The enzyme activity and transcriptional levels of lactate dehydrogenase (LDH) were up-regulated by SCMV infection, and LDH is essential for robust SCMV infection. Moreover, LDH relocates in viral replicase complexes (VRCs) by interacting with SCMV-encoded 6K2 protein, a key protein responsible for inducing VRCs. Additionally, lactate could promote SCMV infection by suppressing plant defense responses. Taken together, we have revealed a viral strategy to manipulate host metabolism to support replication compartment but also depress the defense response during the process of infection.

## Introduction

For successful infection, viruses modify cellular processes and resources to survive and multiply. With the advent of metabolomics, a great deal of information about system-wide changes in plant metabolism during viral infection has become available (Lopez-Gresa et al., 2012; Mandal et al., 2012; Sade et al., 2014). Modulation of host metabolism could benefit viral infection. For example, viruses can obtain energy by activating the host respiration pathway, or build replication compartments by modifying host lipid metabolism to establish a systemic infection in host plants (Sharma et al., 2010; Rosenwasser et al., 2014). In contrast, activation of some metabolic pathways, such as polyamine metabolism, which transduces defense responses, can initiate plant resistance to viral infection (Mitsuya et al., 2009; Sagor et al., 2012). Nonetheless, few studies have explored either changes in host metabolism in response to different stages of viral infection or the metabolites and metabolic pathways that play key roles in the effective process of viral infection.

Upon viral infection, glycolysis is often reprogrammed to meet viral energy needs and provide molecular building blocks (Sharma et al., 2010; Chuang et al., 2017; Nagy and Lin, 2020). During glycolysis, glucose is metabolized to pyruvate, which can be further metabolized by two pathways: (i) under aerobic conditions, pyruvate can be processed by the pyruvate dehydrogenase complex *via* the tricarboxylic acid cycle (TCA) in mitochondria to produce acetyl-COA (Meyer et al., 2019); (ii) under anaerobic conditions, pyruvate can generate ethanol through the concerted actions of pyruvate decarboxylase (PDC) and alcohol dehydrogenase (ADH), or can serve as a substrate for lactate dehydrogenase (LDH) to generate lactate (Dolferus et al., 1997). Recently, enzymes in ethanol fermentation were found to have a positive role in viral infection (Lin et al., 2019). Meanwhile, a recent study demonstrated the importance of lactate in the regulation of animal immunity (Zhou et al., 2021). It will be interesting to test whether enzymes in the lactate fermentation are involved in viral infection. Moreover, the function of lactate in plant immunity deserves further exploration.

In host cells, the virus creates viral replicase complexes (VRCs) to support its robust replication through membrane modification and proliferation, relocation of transport vesicles, and recruitment of a large number of host proteins (Nagy and Pogany, 2011; de Castro et al., 2013; Altan-Bonnet, 2017). Infections by some positive single-stranded RNA viruses are capable of hijacking multiple host-encoded proteins (host factors) to VRCs, and several of which perform a variety of roles throughout the successful viral infection process (Wang, 2015; Hyodo and Okuno, 2016). In addition to helping promote viral replication and establish optimum infection susceptibility conditions, interactions between viral and cellular factors also have impact on host physiological processes (Pallas and Garcia, 2011; Li et al., 2022; Yang et al., 2022). However, though a large number of host proteins that are hijacked by viruses have been identified, few studies have examined the effect of the host-virus interactions on cellular metabolism.

Members of the *Potyvirus* genus have single-stranded, positive-sense RNA genome of approximately 10,000 nucleotides encoding two polyproteins that produce 11 mature proteins by self-cleavage (Chung et al., 2008; Cheng et al., 2017). The *Potyvirus*-encoded 6K2 protein is crucial for the replication and intercellular movement of the viral genome, and also can transport the VRC by inducing the formation of vesicles (Cotton et al., 2009; Wei et al., 2010). Sugarcane mosaic virus (SCMV) is the representative monocot-infecting member of the genus *Potyvirus*, family *Potyviridae*. As the main causal agent of maize dwarf mosaic disease in China and Europe (Jiang and Zhou, 2002; Fan et al., 2003; Jiao et al., 2022), SCMV can also infect sugarcane, sorghum and certain other graminaceous species (Alegria et al., 2003; Achon et al., 2007). Once mosaic symptoms appear on SCMV-infected plants, photosynthesis is significantly inhibited (Chen et al., 2017a; Akbar et al., 2020).

In this study, we found through metabolomics analysis that lactate accumulated immediately before and after robust SCMV infection. Induction of lactate-involved anaerobic glycolysis promotes SCMV infection. The function of the LDH enzyme in the lactate fermentation pathway is essential for effective SCMV infection. LDH is recruited into the VRCs by interacting with SCMV-encoded 6K2. In addition, we determined that lactate can suppress plant defense responses to promote SCMV infection. Altogether, our results elucidate the multiple mechanisms through which viruses manipulate host metabolism for successful infection.

## Material and methods

### Plant growth and virus inoculation

Maize (*Zea mays*) inbred line B73 and *Nicotiana benthamiana* plants were grown growth in chambers maintained at 24/22°C (day/night) and a 16/8 h (light/dark) photoperiod. SCMV Beijing strain (SCMV-BJ) was from a previously published source (Fan et al., 2003). *N. benthamiana* leaves were infiltrated with *Agrobacterium tumefaciens* (at an OD_600_ of 1.5) carrying SCMV infectious clones. Crude extracts from the SCMV-BJ infected maize leaves or SCMV infectious clone-infected *N. benthamiana* leaves were used to rub-inoculate maize seedling young-expanded leaves as described previously (Chen et al., 2017a).

### Total RNA extraction and gene expression analysis

Total RNA was extracted from individual leaf samples using TRIzol reagent followed by RNase-free DNase I treatment. The cDNA synthesis was performed using 2 μg total RNA per sample, an oligo(dT_18_) primer, and M-MLV reverse transcriptase in a 25 μL reaction. Quantitative reverse transcription-polymerase chain reaction (RT-qPCR) was performed using 1 μL of 10-fold diluted cDNA per reaction, gene-specific primers (**Supplementary Table 2**), and a Fast SYBR mixture on an ABI 7500 Real Time PCR system (Applied Biosystems Inc.). The expression level of the maize *ubiquitin* gene (*ZmUbi*) was used as the internal control (Chen et al., 2017a). The relative expression levels of the assayed genes were calculated using the 2^-ΔΔCT^ method (Livak and Schmittgen, 2001).

### Western blotting analysis

Total protein was isolated from individual leaf samples and separated in gels through electrophoresis as previously described (Cao et al., 2012). Detections of specific proteins on the immunoblots were performed using antibodies specific for SCMV coat protein (CP) (Xia et al., 2016), Flag (Sigma-Aldrich) and plant β-actin (EASYBIO). The relative expression levels of individual proteins on the immunoblots were quantified using the ImageJ image analysis tool (http://imagej.net/) as previously described (Wyrsch et al., 2015).

### Metabolomics

At 4- and 5-day post inoculation (dpi), mock-inoculated and SCMV-infected leaf samples from six independent biological replicates were collected. To minimize the effect of variation in metabolite content throughout the plant, maize leaves from SCMV-infected plants and the corresponding mock-inoculated plants were harvested at the same leaf position. Collected tissue was immediately frozen in liquid nitrogen and stored at -80°C until further analysis. Maize leaf powder was weighed to 50 mg and extracted with 450 μL extraction liquid (75% methanol). The mixture was homogenized in a ball mill for 4 min at 45 Hz and then ultrasonically treated for 5 min (incubated in ice water); this process was repeated 3 times. The supernatant was collected after centrifugation at 12,000 rpm for 15 min. The sample extract was filtered through a 0.22 µm filter and added to sample vials. All samples were analysed by a gas chromatograph system coupled with a Pegasus HT time-of-flight mass spectrometer (GC-TOF-MS). The GC-TOF-MS analysis was performed at Shanghai Applied Protein Technology Co. Ltd. and was conducted with an Agilent 1290 Infinity chromatography system and AB SCIEX QTRAP 5500 mass spectrometer. MultiQuant software was used to extract the chromatographic peak area and retention time. The AA standard correct retention time was used to identify the metabolites. To identify the differentially expressed metabolites, statistical analyses between the two sample groups were performed by calculating the variable importance in the projection (VIP) and *p* values of the metabolites. Student’s *t* test was used to obtain *p* values. Metabolites with VIP > 1 and *p* values < 0.05 were marked as differentially expressed metabolites between sample groups. Pathway enrichment analysis was performed using MetaboAnalyst 5.0 (www.metaboanalyst.ca/MetaboAnalyst/).

### Lactate measurement

Fine powder from leaf samples (~50 mg) was mixed with 600 μL of acetonitrile: chloroform (7: 3, v/v) solution and vortexed for 30 s. The quantitation control was prepared by adding 20 ng L-lactate (Sigma−Aldrich, L1750) into the acetonitrile: chloroform solution. The mixture was sonicated for 1 h on ice and then centrifuged for 5 min at 7,000 g at 4°C. Three hundred microlitres of H_2_O was added to each supernatant prior to the two-step liquid−liquid partitioning. The upper aqueous fractions from the same sample were pooled and dried under a nitrogen stream. The dried extracts were then dissolved in 750 μL of H_2_O and filtered through a 0.22 μm membrane prior to measurement. LC–MS/MS analysis was performed on a UPLC system (Waters, Milford, Ohio, USA) combined with a 5500 Qtrap MS equipped with an ESI source (AB SCIEX). Each sample (5 μL) was injected onto an HSS T3 C18 column for further analysis.

### Chemical agent treatment

Maize plants were separately sprayed with 100 nM UK5099 (Sigma−Aldrich; dissolved in DMSO), 100 μM DCA (Sigma−Aldrich; dissolved in double-distilled water), or 10 μM lactate (Sigma−Aldrich; dissolved in double-distilled water) containing 0.2% Tween-20, or with a 0.2% Tween-20 solution with no chemical agents as a blank control.

### Sequence accessions and sequence alignment analysis

Putative *ZmLDH1* and *ZmLDH2* genes were obtained from the updated maize B73 genome website (AGPv4, http://ensembl.gramene.org/Zea_mays/Info/Index). Multiple sequence alignments were performed by DNAMAN 7.0 (Lynnon Biosoft, San Ramon, CA, USA).

### LDH enzymatic activity

Extractions were carried out on ice. Maize leaves were ground in extraction buffer (0.1 M Tris-HCl, pH 8.5, 10 mM Na borate, 10 mM DTT and 5 mg/ml BSA). The sample was centrifuged, and aliquots of the supernatant were taken for the enzyme assay. LDH enzymatic activity was assayed by monitoring the pyruvate-dependent NADH oxidation based on the absorbance at 340 nm spectrophotometrically. The assay mix (final volume 1.3 ml) contained 1 ml of 0.13 M Tris-HCl (pH8.0), 150 μg NADH, 3 μmol 4-methylpyrazole, 3 μmol NaCN, 15 μmol Na pyruvate, and 0.2 ml of enzyme extract.

### Plasmid construction

The pGD-6K2-VPg-Pro-mCherry vector used in this study was described previously (Xie et al., 2021). For transient expression assays in maize protoplasts, coding region sequence (CDS) of *ZmLDH2* was cloned into the pGD-eGFP vector. For overexpression *via* SCMV, the LDH2_3Flag_ and GFP_3Flag_ fragments replaced the GFP fragment of pSCMV-GFP to produce pSCMV-LDH2_3Flag_ and pSCMV-GFP_3Flag_, respectively. For the luciferase complementation imaging (LCI) assays, CDS of *ZmLDH2* was inserted into pCAMBIA-Cluc vectors at the *Kpn* I and *Sal* I sites to generate pUC-CE-ZmLDH2; and *6K2* was inserted into pCAMBIA-Nluc vectors at the *Sac* I and *Sal* I sites to generate pUC-NE-6K2, respectively. For the biomolecular fluorescence complementation (BiFC) assays, ZmLDH2 and 6K2 were inserted into pUC-CE or pUC-NE vectors at the *BamH* I and *Sal* I sites to generate pUC-CE-ZmLDH2 and pUC-NE-6K2, respectively. For cucumber mosaic virus (CMV)-virus-induced gene silencing (VIGS) assessment, a 200 bp DNA fragment representing a conserved partial sequence of *ZmLDH1* and *ZmLDH2* was amplified *via* RT-PCR using specific primers (**Supplementary Table 2**). The resulting fragment was cloned into the pCMV201-2b_N81_ vector resulted in pCMV201-2b_N81_:LDH. All constructs were checked by sequencing prior to use.

### CMV-based gene silencing in maize

Agrobacterium cultures carrying pCMV101, pCMV301 or one of the two constructs (pCMV201-2b_N81_:LDH, and pCMV201-2b_N81_:GFP) were grown, mixed, and infiltrated into the leaves of *N. benthamiana* plants as described (Wang et al., 2016). At 4 days post infiltration, the infiltrated *N. benthamiana* leaves were processed into crude leaf extracts, which were then sap-inoculated individually into maize seedlings using the vascular puncture inoculation (VPI) method (Wang et al., 2016).

### Maize protoplasts isolation and transfection

Maize seeds were inoculated with crude leaf extracts from SCMV-BJ-infected or noninfected (control) maize plants using the VPI method. The germinated seedlings were kept in the dark at 24°C to produce etiolated plants. Maize protoplasts isolation and transfection were performed as described (Zhu et al., 2014).

At 14 hours post transfection, maize protoplasts were examined with a Zeiss LSM 800 confocal microscope for subcellular localization assays. For EGFP, the excitation wavelength was set at 488 nm and the emission wavelength at 510–550 nm. For mCherry protein, the excitation wavelength was set at 552 nm and the emission wavelength at 562–632 nm.

### Yeast two-hybrid

The yeast two-hybrid (Y2H) assay was performed in accordance with the procedures provided by the manufacturer (Clontech). Yeast expression plasmids were introduced into the yeast strain Gold (Clontech), and all transformants were cultured at 30°C for 72 h on synthetic dextrose (SD) medium without Leu and Trp. Afterwards, they were switched to a medium lacking Leu, Trp, His, and Ade.

### LCI

LCI assays were performed as previously described (Chen et al., 2008). All of the tested combinations were agroinfiltrated into *N. benthamiana* leaves. At 3 days after infiltration, the leaves were sampled, sprayed with 1 mM luciferin (Invitrogen), and photographed using a low-light cooled CCD imaging apparatus (iXon, Andor Technology, Belfast, UK). The pictures were taken 15 min after exposure.

### BiFC assays

Maize protoplasts were transfected with different combinations of expression vectors. At 14 h post-transfection, YFP was excited at 514 nm with emission detected at 565–585 nm using a Zeiss LSM 800 confocal microscope.

### Measurement of H_2_O_2_ in maize leaves

Measurement of H_2_O_2_ content in assayed maize leaves was performed using the Amplex Red Hydrogen Peroxide/Peroxidase Assay Kit (Invitrogen, Carlsbad, USA) as instructed. Fluorescence was excited at a wavelength of 530 nm and detected at a wavelength of 590 nm.

Maize leaf tissues were cut and incubated for 10 h in a water solution supplemented with 10 nM CM-H_2_DCFDA [5-(and-6)-chloromethyl 2’,7’-dichlorodihydrofluorescein diacetate, Invitrogen] to measure H_2_O_2_ levels and then washed twice before imaging.

### Statistical analyses

The statistical significance of the data was determined by GraphPad Prism 7.0 (GraphPad Software Inc., USA: http://www.graphpad.com/). Comparisons between two groups of data were calculated by Student’s *t*-test or ANOVA (*P<0.05; **P<0.01; ***P<0.001).

## Results

### Untargeted metabolomics analysis showed lactate accumulation immediately before and after robust SCMV infection

**Figure 1:**
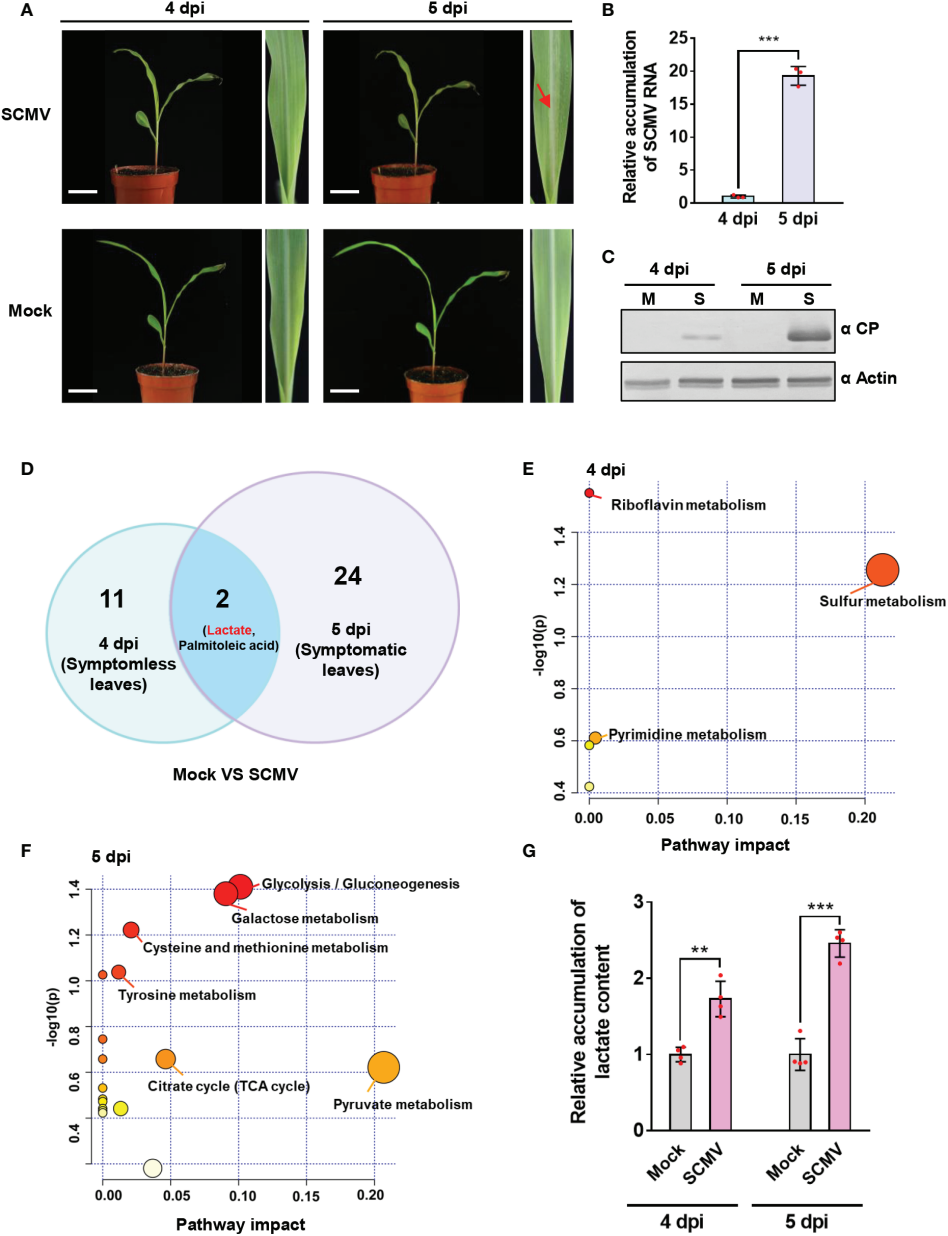
Untargeted metabolomics analysis showed significant lactate accumulation at 4- and 5-day post inoculation (dpi) of sugarcane mosaic virus (SCMV). **(A)** Maize plants and the first systemically infected maize leaves (1 SLs) of mock-inoculated or SCMV-infected maize plants at 4 and 5 dpi. Maize leaves (n=6) were harvested, pooled, and used for untargeted metabolomics analysis. Red arrow indicates the manifestation of mosaic symptoms. Scale bars = 4 cm. **(B)** Relative accumulation levels of SCMV genomic RNA in 1 SLs determined by RT-qPCR at 4 and 5 dpi. The results are represented as the means ± SE (n=3). Statistical differences between the treatments were determined using unpaired Student’s *t*-test (two-tailed), ***P < 0.001. **(C)** Accumulation levels of SCMV coat protein (CP) in 1 SLs at 4 and 5 dpi through western blotting analysis. The actin bands in the lower panels are used to show sample loadings. M, Mock; S, SCMV. **(D)** Venn diagram displaying the numbers of differentially expressed metabolites identified at 4 dpi and 5 dpi. Lactate and palmitoleic acid were found in both groups. **(E)** Pathway enrichment analysis (GlobalTest) combined with pathway topology analysis (degree centrality and betweenness centrality) for SCMV-infected plants compared with mock-inoculated plants at 4 dpi. Metabolites are clustered in nodes with pathway impact on the X axis calculated by pathway topology analysis and plotted according to –log (*p*) values on the Y axis. SCMV-infected plants demonstrated multiple large nodes of altered metabolic pathways compared with mock-inoculated plants. **(F)** Pathway enrichment analysis (GlobalTest) combined with pathway topology analysis (degree centrality and betweenness centrality) for SCMV-infected plants compared with mock-inoculated plants at 5 dpi. **(G)** Relative accumulation of lactate contents in the Mock- or SCMV-infected samples harvested at 4 or 5 dpi through LC-MS/MS. The data are represented as the means ± SE (n=4). Statistical differences between the treatments were determined using unpaired Student’s *t*-test (two-tailed), **P < 0.01; ***P < 0.001.

Previous studies found that maize plants mainly showed systemically mosaic symptoms at 5 dpi of SCMV (Chen et al., 2017a; Chen et al., 2017b; Du et al., 2020). In this study, we also discovered that only 1.3% of maize plants (3 of 221-inoculated plants in three replicates) started to show mosaic symptoms at 4 dpi, whereas almost all SCMV-infected maize plants developed mosaic symptoms on the first systemically infected leaf (referred to as 1 SL) at 5 dpi (**Figure 1** and **Supplementary Figure 1**). RT-qPCR and western blotting showed the quite low accumulation levels of SCMV genomic RNA and CP at 4 dpi while their dramatical increase at 5 dpi (**Figure 1C**). Therefore, we determined that the time from 4 to 5 dpi was a key period for robust SCMV multiplication and infection.

To elucidate the metabolites and metabolic pathways that might be crucial for effective SCMV infection, we sampled maize leaves at two time points for untargeted metabolomics analysis. One was before the manifestation of mosaic symptoms (on 4 dpi), and another was immediately after the appearance of symptoms (on 5 dpi). Results of metabolomics analyses revealed that thirteen metabolites showed significant changes (VIP > 1, *p* < 0.05) at 4 dpi (**Figure 1** and **Supplementary Table 1**). Topological analysis revealed that the functions of the 13 differentially expressed metabolites at 4 dpi were mainly enriched in sulfur metabolism and riboflavin metabolism (**Figure 1**). Thus, SCMV infection could affect the biosynthesis of sulfur-containing defense compounds and antioxidant compounds before symptoms appearance, both of which involved in plant resistance (Gao et al., 2012; Deng et al., 2014; Zhao et al., 2014). At 5 dpi, with the manifestation of mosaic symptoms and robust multiplication of SCMV in maize leaves, 26 metabolites were significantly changed (**Figure 1** and **Supplementary Table 1**). Topological analysis revealed that the differentially expressed metabolites at 5 dpi were primarily linked to pyruvate metabolism, glycolysis, and the TCA cycle (**Figure 1**). Thus, SCMV infection at this stage primarily affected plant energy metabolism. Notably, both lactate and palmitoleic acid changed significantly among all the differentially expressed metabolites in both periods (**Figure 1**). We further, by using LC−MS/MS, confirmed that lactate accumulated much higher levels in SCMV-infected plants, i.e. 1.7- and 2.4-fold higher at 4 and 5 dpi, respectively, than those in mock-inoculated plants (**Figure 1**). These results indicate that lactate accumulation may play an essential role in supporting robust SCMV infection.

### Induction of lactate-involved anaerobic glycolysis promotes SCMV infection

**Figure 2:**
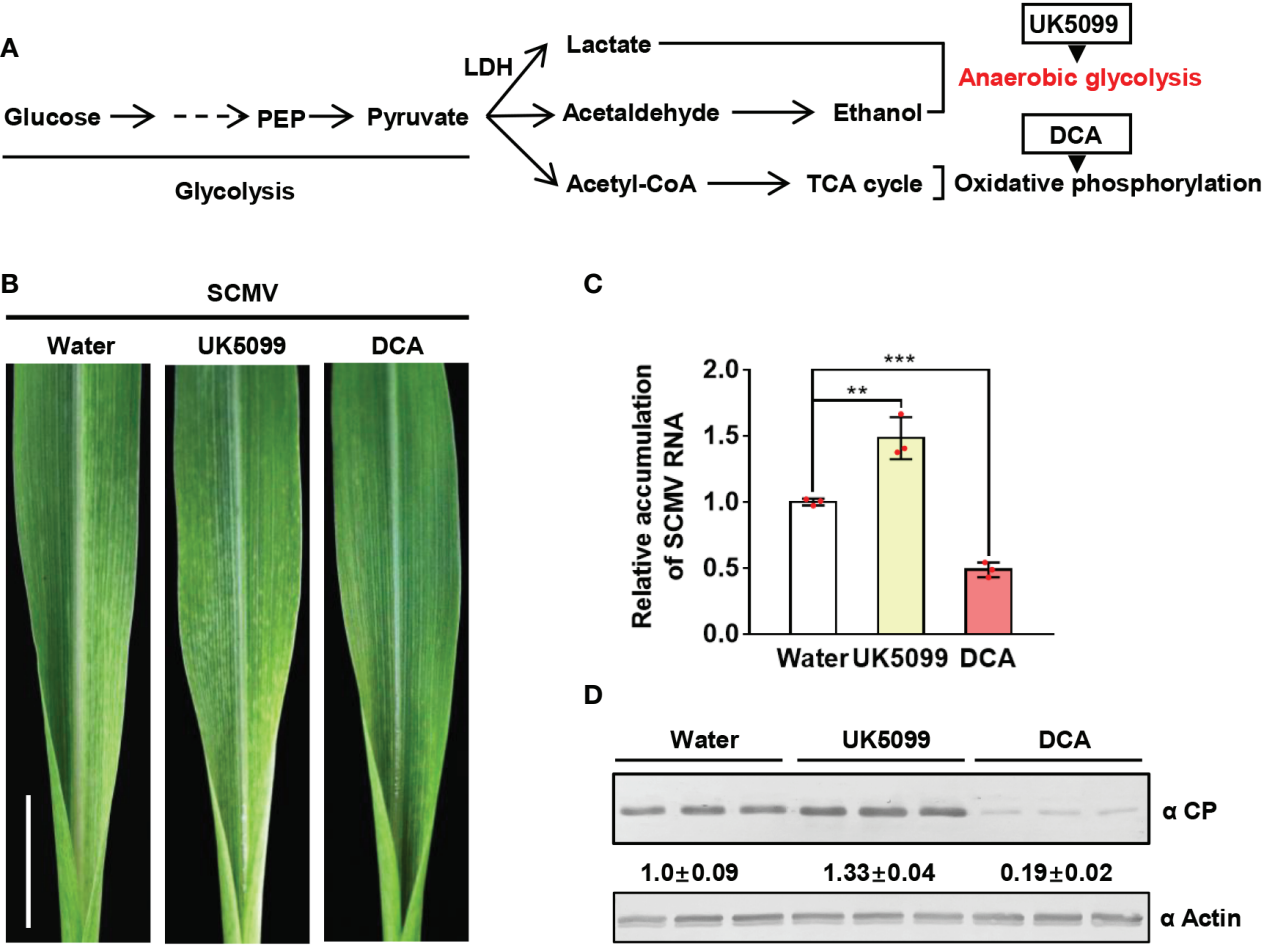
Induction of anaerobic glycolysis promotes SCMV infection. **(A)** Simplified scheme of the glucose metabolic pathway. The end product of glycolysis, pyruvate, is further metabolized to lactate and alcohol in the anaerobic metabolic pathway or to acetyl-CoA in the oxidative phosphorylation pathway for the TCA cycle. UK5099 and DCA were used to induce anaerobic glycolysis and oxidative phosphorylation, respectively. **(B)** Mosaic symptoms in UK5099-treated plants were more severe than that of water-treated plants, whereas those in DCA-treated plants were milder than in water-treated plants. Scale bars = 1 cm. **(C)** Relative accumulation levels of SCMV genomic RNA, determined by RT-qPCR, in 1 SLs of the water-, UK5099- or DCA-treated plants at 5 dpi. The results are represented as the means ± SE (n=3). Statistical differences between the treatments were determined using unpaired Student’s *t*-test (two-tailed), **P < 0.01; ***P < 0.001. **(D)** Accumulation levels of SCMV CP in 1 SLs of the water-, UK5099- or DCA-treated plants at 5 dpi through western blotting analysis. The samples harvested from the water-treated plants were used as controls. The detected protein bands were visualized using the ImageJ software. The numbers between the two panels are the relative ratios of SCMV CP accumulated in UK5099- or DCA-treated plants verses the control plants. The amount of SCMV CP in the control plants is arbitrarily presented as 1.0. The actin bands in the lower panels are used to show sample loadings.

Since lactate is a product of anaerobic glycolysis, we firstly determined whether anaerobic glycolysis plays a role in SCMV infection. Oxidative phosphorylation (OxPhos) and anaerobic glycolysis are the two major catabolic glucose pathways in plants (**Figure 2**). To dissect the key step of glucose metabolism involved in SCMV infection, we treated SCMV-infected maize plants once at 3 dpi with UK5099, which is known to promote anaerobic glycolysis, or dichloroacetate (DCA), which controls the anaerobic glycolysis shift to OxPhos (Zhou et al., 2021). By 5 dpi, UK5099 treatment of SCMV-infected maize plants caused more severe mosaic symptoms on maize leaves compared with that water-treated (**Figure 2**). Both RT-qPCR and western blotting results showed significant increment of SCMV RNA and CP by UK5099 treatment (**Figure 2D**). In contrast, treatment with DCA alleviated the severity of mosaic symptoms on maize leaves, and decreased SCMV RNA and CP accumulation levels compared with that water-treated (**Figure 2D**). Taken together, induction of anaerobic glycolysis promotes SCMV infection.

### SCMV infection up-regulates the enzyme activity of LDH

**Figure 3:**
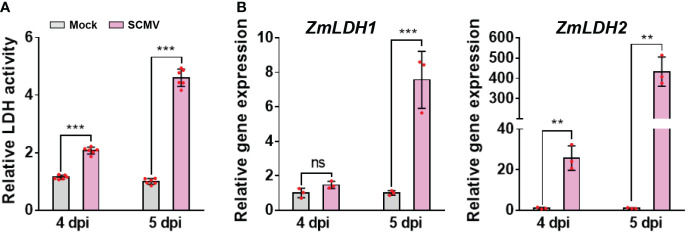
SCMV infection up-regulates LDH activity and genes expression. **(A)** Analysis of LDH enzyme activities in 1 SLs of mock-inoculated or SCMV-infected plants at 4 and 5 dpi. The results are represented as the means ± SE (n=6). **(B)** Relative transcriptional expression levels of *ZmLDH1* and *ZmLDH2* determined by RT-qPCR using mock-inoculated or SCMV-infected leaves harvested at 4 and 5 dpi. The data are represented as the means ± SE (n=3). Statistical differences were determined using unpaired Student’s *t*-test (two-tailed), ns, no significance; **P < 0.01; ***P < 0.001.

In the lactate fermentation pathway of anaerobic glycolysis, lactate is produced from pyruvate by cytosolic NAD-dependent LDH (Maurino and Engqvist, 2015). Here, we determined whether LDH participates in SCMV infection. We found that the enzymatic activity of LDH was significantly up-regulated immediately before and after robust SCMV infection, i.e. at 4 and 5 dpi (**Figure 3**).

Next, we analysed the sequences of *ZmLDH* genes and then investigated their transcriptional patterns following SCMV infection. Two homologs of maize *LDH* were obtained from the updated *Z. mays* B73 genome (AGPv4, http://ensembl.gramene.org/Zea_mays/Info/Index): *ZmLDH1* (Zm00001d014944) and *ZmLDH2* (Zm00001d014945). Sequence alignments indicated that the two *LDH* genes shared approximately 90% and 89% identities on nucleotide and amino acid sequences, respectively (**Supplementary Figure 2A**). We used RT-qPCR to determine the relative expression levels of different *ZmLDH* transcripts in mock-inoculated and SCMV-infected maize plants. At 4 dpi, the expression level of *ZmLDH1* was not significantly changed compared with that of mock-inoculated plants, while the expression level of *ZmLDH2* was strongly up-regulated 40-fold by SCMV infection (**Figure 3**). At 5 dpi, the expression levels of *ZmLDH1* and *ZmLDH2* in systemically infected leaves were approximately 7.5-fold and 400-fold higher, respectively, than that in the equivalent leaves of mock-inoculated plants (**Figure 3**). These results suggest that LDH might play important roles for SCMV infection. Considering the high identities between ZmLDH1 and ZmLDH2 and the much higher expressional change of *ZmLDH2* caused by SCMV infection than that of *ZmLDH1*, subsequently, we chose ZmLDH2 for the following studies.

### ZmLDH is essential for robust SCMV infection

**Figure 4:**
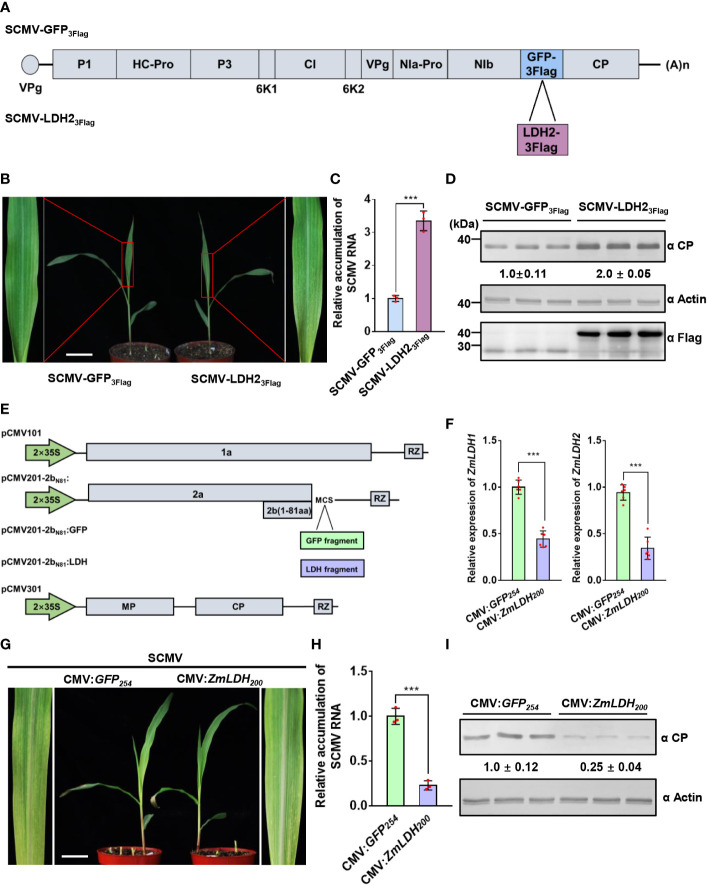
ZmLDH is essential for robust SCMV infection. **(A)** Schematic representation of SCMV-related constructs. GFP_3Flag_ or LDH2_3Flag_ is inserted between NIb and CP to obtain SCMV-GFP_3Flag_ or SCMV-LDH2_3Flag_. **(B)** Mosaic symptoms in SCMV-LDH2_3Flag_-infected plants were more severe than that in SCMV-GFP_3Flag_-infected plants. Scale bars = 4 cm. **(C)** Relative accumulation levels of SCMV genomic RNA, determined by RT-qPCR, in 1 SLs of SCMV-GFP_3Flag_- or SCMV-LDH2_3Flag_-infected plants at 5 dpi. The results are represented as the means ± SE (n=3). Statistical differences between the treatments were determined using unpaired Student’s *t*-test (two-tailed), ***P < 0.001. **(D)** Detection of SCMV CP, GFP_3Flag_, and LDH2_3Flag_ in 1 SLs of SCMV-GFP_3Flag_- or SCMV-LDH2_3Flag_-infected plants at 5 dpi through western blotting analysis. The samples harvested from the SCMV-GFP_3Flag_-infected plants were used as controls. The detected protein bands were visualized using the ImageJ software. The numbers between the two panels are the relative ratios of SCMV CP accumulated in the SCMV-LDH2_3Flag_-infected plants verses the control plants. The amount of SCMV CP in the control plants is arbitrarily presented as 1.0. The actin bands in the lower panels are used to show sample loadings. **(E)** Schematic representation of ZMBJ-CMV-based gene silencing vector pCMV101, pCMV201-2b_N81_ and pCMV301. Fragments of *GFP* and *LDH* were separately cloned into pCMV201-2b_N81_ vector to result in pCMV201-2b_N81_:GFP and pCMV201-2b_N81_:LDH. **(F)** Expression levels of *ZmLDH1* and *ZmLDH2* were analyzed through RT-qPCR using gene specific primers. Data are represented as the means ± SE (n=6). Statistical differences between the treatments were determined using unpaired Student’s *t*-test (two-tailed), ***P < 0.001. **(G)** Silencing of *ZmLDH* expression in maize with CMV vector alleviated SCMV mosaic symptoms in maize leaves. The maize plants inoculated with CMV : GFP_254_ were used as the controls. All maize leaves were photographed at 7 dpi of SCMV infection. Scale bars = 4 cm. **(H)** Relative accumulation levels of SCMV genomic RNA, determined by RT-qPCR, in 1 SLs of the control or *ZmLDH*-silenced plants at 7 dpi. The results are represented as the means ± SE (n=3). Statistical differences between the treatments were determined using unpaired Student’s *t*-test (two-tailed), ***P < 0.001. **(I)** Accumulation levels of SCMV CP in the systemically infected leaves harvested from the control or *ZmLDH*-silenced plants at 7 dpi through western blotting analysis. The samples harvested from the CMV : GFP_254_-inoculated plants were used as controls. The detected protein bands were visualized using the ImageJ software. The numbers between the two panels are the relative ratios of SCMV CP accumulated in the *ZmLDH*-silenced plants verses the control plants. The amount of SCMV CP in the control plants is arbitrarily presented as 1.0. The actin bands in the lower panels are used to show sample loadings.

To explore the role of LDH in viral infection, we overexpressed *ZmLDH2* using an LDH2-expressing SCMV infectious clone designated pSCMV-LDH2_3Flag_, with pSCMV-GFP_3Flag_ as a control (**Figure 4**). We used the sap of *N. benthamiana* leaves agroinfiltrated with each of these two virus infectious clones to mechanically inoculate maize plants. SCMV-LDH2_3Flag_-infected plants showed mosaic symptoms (4 dpi) earlier than SCMV-GFP_3Flag_-infected plants (5 dpi) (**Supplementary Figure 3**). At 5 dpi, maize plants infected by SCMV-LDH2_3Flag_ exhibited more severe mosaic symptoms on 1 SLs than that of SCMV-GFP_3Flag_-infected plants (**Figure 4**). The accumulation of SCMV genomic RNA in the SCMV-LDH2_3Flag_-infected plants increased by 3.3-fold at 5 dpi compared with that in SCMV-GFP_3Flag_-infected plants (**Figure 4**). Western blotting results were consistent with the RT-qPCR results and showed 2.0-fold increases of SCMV CP accumulation at 5 dpi (**Figure 4**). Detection of the expression of Flag-tagged proteins in systemically infected leaves by western blotting demonstrates that both SCMV-LDH2_3Flag_ and SCMV-GFP_3Flag_ can successfully infect maize plants (**Figure 4**). These results show that overexpression of *ZmLDH2* enhances SCMV infection.

Meanwhile, we silenced both *ZmLDH1* and *ZmLDH2* using the ZMBJ-CMV-based gene silencing vector (Wang et al., 2016). A DNA fragment (200 bp) that is conserved between *ZmLDH1* and ZmLDH2 was selected after analysis for minimal off-target silencing, and then was cloned into pCMV201-2b_N81_ (**Figure 4**). Maize B73 seeds were inoculated with ZMBJ-CMV harboring the *ZmLDH_200_* or *GFP_254_* fragment using VPI (Wang et al., 2016). Maize plants inoculated with CMV-GFP_254_ were used as controls. RT-qPCR results showed that the relative expression of *ZmLDH1* and *ZmLDH2* reduced *c.* 56 and 66%, respectively, in silenced plants at 7 days post-SCMV infection compared with the CMV : GFP control plants (**Figure 4**). Silencing of *ZmLDH* expression did not affect maize growth, and milder, later (7 dpi) mosaic symptoms were observed in the *ZmLDH-*silenced plants than in the non-silenced control plants (5 dpi) (**Figure 4** and **Supplementary Figure 3**). Meanwhile, SCMV genomic RNA levels in the *ZmLDH*-silenced plants decreased by *c.* 78% compared with those in the control plants (**Figure 4**). SCMV CP accumulation also reduced by 75% in the *ZmLDH*-silenced plants compared with that in the control plants (**Figure 4**). Taken together, expression of *ZmLDH* is essential for robust SCMV infection.

### SCMV encoded 6K2 interacts with ZmLDH2

**Figure 5:**
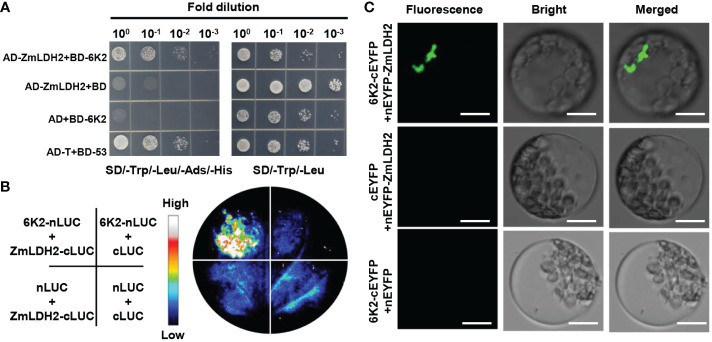
SCMV-encoded 6K2 interacts with ZmLDH2 *in vitro* and *in vivo*. **(A)** Analysis of the interaction between 6K2 and ZmLDH2 by yeast two-hybrid assays. Serial dilutions of yeast cells co-transfected with two recombination vectors were plated on SD–Trp–Leu–His–Ade medium. Yeast cells co-transfected with pGADT7-T (AD-T) and pGBKT7-p53 (BD-53) were used as positive controls. **(B)** Analysis of the interaction between 6K2 and ZmLDH2 by luciferase complementation imaging (LCI) assays. The *Agrobacterium* strains carrying the indicated constructs were infiltrated into *N. benthamiana* leaves. Luciferase activities were recorded 3 days after infiltration. **(C)** Biomolecular fluorescence complementation (BiFC) analysis of the interaction between 6K2 and ZmLDH2. ZmLDH2 and 6K2 fused to N or C-terminus of YFP were transiently co-expressed in maize protoplasts. Confocal analysis was performed at 14 h post transfection. Representative results of at least three independent experiments are shown. Scale bars = 20 µm.

To explore the mechanism by which ZmLDH promotes SCMV infection, we used Y2H arrays to investigate potential interactions between SCMV-encoded proteins and LDH2. The Y2H analysis showed that 6K2 interacted with ZmLDH2 (**Figure 5**). To further confirm the interaction between 6K2 and ZmLDH2, we performed LCI and BiFC assays. For the LCI assay, the restoration of luciferase activity by the 6K2-ZmLDH2 interaction led to the detection of a positive luciferase signal in the leaf area co-expressing 6K2 and ZmLDH2. Negative controls showed no luciferase signal (**Figure 5**). For the BiFC assay, N-terminal YFP-tagged 6K2 (6K2-nYFP) and C-terminal YFP-tagged LDH2 (cYFP-LDH2) were co-expressed in maize protoplasts. Aggregated YFP fluorescence (positive interaction signals) were observed only in maize protoplasts co-expressing 6K2 and ZmLDH2 (**Figure 5**). Together, these results suggest that SCMV 6K2 interacts with ZmLDH2 in yeast and in planta.

### ZmLDH2 re-localizes to the SCMV replication complex

**Figure 6:**
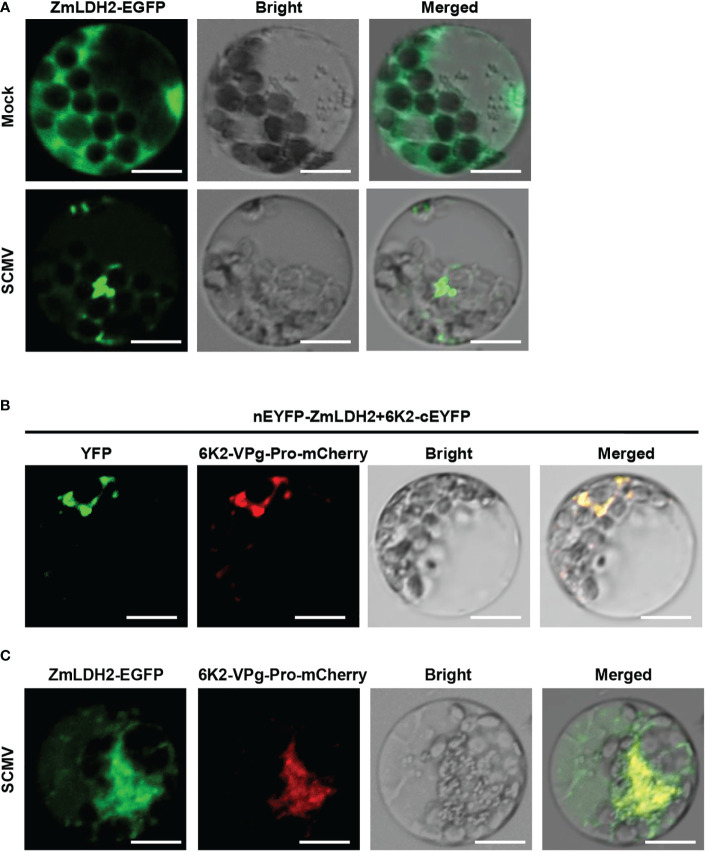
Subcellular colocalization of ZmLDH2 with SCMV viral replicase complexes (VRCs) in maize protoplasts. **(A)** Subcellular localization of ZmLDH2-GFP in mock-transfected or SCMV-infected maize protoplasts. Images were taken at 14 h post transfection. Scale bars = 20 µm. **(B)** Co-localization assay of BiFC signals with the SCMV VRCs marker 6K2-VPg-Pro-mCherry in maize protoplasts. Images were taken at 14 h post transfection. Scale bars = 20 µm. **(C)** Co-localization of ZmLDH2-GFP with the SCMV VRCs marker 6K2-VPg-Pro-mCherry in SCMV-infected maize protoplasts. Images were taken at 14 h post transfection. Scale bars = 20 µm.

Since 6K2 is a key protein that induces the formation of the ER-derived vesicles for potyviruses replication (Wei et al., 2010), we determined whether ZmLDH2 is recruited into the VRCs of SCMV. Transiently single expression of ZmLDH2-GFP localized to the cytoplasm in maize protoplasts (**Figure 6**). In contrast, in SCMV-infected cells, ZmLDH2 aggregated in the cytoplasm (**Figure 6**). To identify the precise compartment for the interaction of 6K2 and LDH2, cYFP-ZmLDH2 and 6K2-nYFP were co-expressed with 6K2-VPg-Pro-mCherry, which was previously used to indicate SCMV VRCs (Xie et al., 2021). Confocal microscopy observations showed that the site of the 6K2-ZmLDH2 interaction colocalized with aggregated 6K2-VPg-Pro (**Figure 6**). In addition, the aggregation of ZmLDH2 under SCMV infection also colocalized with aggregated 6K2-VPg-Pro (**Figure 6**). Altogether, ZmLDH2 can be relocated into SCMV VRCs.

### Lactate promotes SCMV infection by inhibiting plant immunity

**Figure 7:**
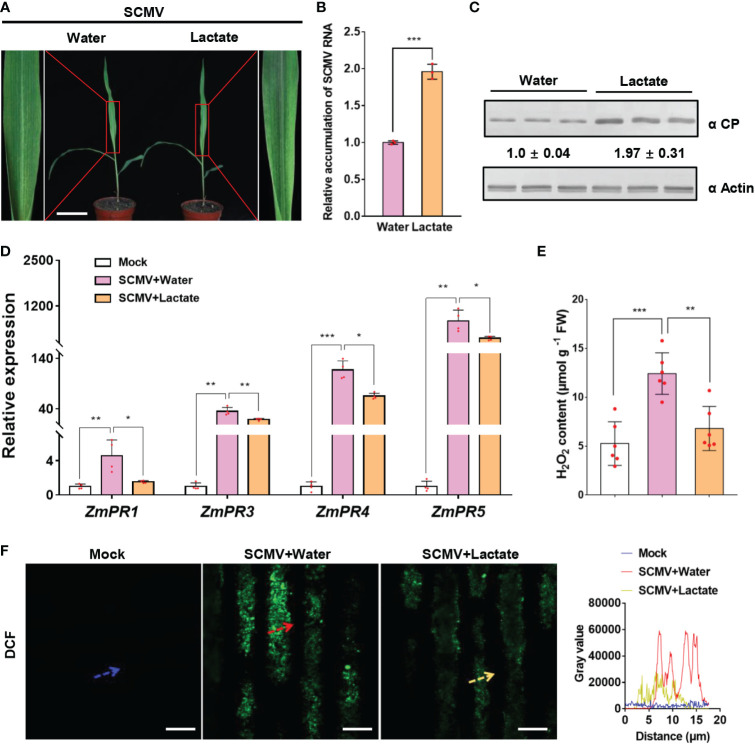
Lactate promotes SCMV infection *via* suppressing plant defense responses. **(A)** Mosaic symptoms in lactate-treated plants were more severe than that in water-treated plants. Scale bars = 8 cm. **(B)** Relative accumulation levels of SCMV genomic RNA, determined by RT-qPCR, in 1 SLs of the water- or lactate- treated plants at 5 dpi. The results are represented as the means ± SE (n=3). Statistical differences between the treatments were determined using unpaired Student’s *t*-test (two-tailed), ***P < 0.001. **(C)** Accumulation levels of SCMV CP in 1 SLs harvested from the water- or lactate-treated plants at 5 dpi through western blotting analysis. The samples harvested from the water-treated plants were used as controls. The detected protein bands were visualized using the ImageJ software. The numbers between the two panels are the relative ratios of SCMV CP accumulated in the lactate-treated plants verses the control plants. The amount of SCMV CP in the control plants is arbitrarily presented as 1.0. The actin bands in the lower panels are used to show sample loadings. **(D)** *PR* genes expression was detected by RT-qPCR in mock-inoculated or SCMV-infected plants treated with water or lactate for 24 h. The data are represented as the means ± SE (n=4). Statistical differences between the treatments were determined using unpaired Student’s *t*-test (two-tailed), *P < 0.05; **P < 0.01; ***P < 0.001. **(E)** Detection of H_2_O_2_ production in mock-inoculated plants or in SCMV-infected maize plants with water or lactate treatment for 24 h. The results are represented as the means ± SE (n=6). Statistical differences between the treatments were determined using unpaired Student’s *t*-test (two-tailed), **P < 0.01; ***P < 0.001. **(F)** Detection of H_2_O_2_ in mock-inoculated maize leaves or in SCMV-infected maize leaves with water or lactate treatment for 24 h. H_2_O_2_ was visualized using the fluorescence probe CM-H_2_DCFDA (DCF; green fluorescence). The relative fluorescence along the dotted line in the photos is represented by the gray value plots. Scale bars = 10 μm.

Lactate has been reported to function in animal immune escape of hepatitis B virus (HBV) infection (Zhou et al., 2021). We next determined whether lactate, the product of LDH catalysis, also functions in SCMV infection by interfering with plant immunity. To evaluate the role of lactate in SCMV infection, we sprayed SCMV-infected maize plants with lactate at 3 dpi. Plants inoculated with SCMV and then sprayed with water were used as controls. By 5 dpi, the lactate-treated and SCMV-infected plants exhibited more severe mosaic symptoms than water-treated control plants (**Figure 7**). The results of RT-qPCR and western blotting analyses showed significantly enhanced accumulation of SCMV genomic RNA and CP in maize plants treated with lactate compared to control plants (**Figure 7C**).

To investigate whether immunity response correlated with enhanced SCMV infection by lactate treatment, we measured the expression levels of the *pathogenesis-related* (*PR*) genes and the accumulation of H_2_O_2_ in SCMV-infected maize leaves at 24 h post lactate or water treatment. As previously reported (Chen et al., 2017a; Yuan et al., 2019), SCMV infection stimulated the expression levels of *PR* genes (*ZmPR1*, *ZmPR3*, *ZmPR4* and *ZmPR5*) and the accumulation levels of H_2_O_2_ (**Figure 7F**). Intriguingly, lactate treatment significantly decreased the expression levels of *PR* genes in SCMV-infected plants (**Figure 7**). Meanwhile, the abundance of H_2_O_2_ and fluorescence intensity of CM-H_2_DCFDA (DCF) were dramatically lowed in lactate-treated maize leaves than that in water-treated maize leaves during SCMV infection (**Figure 7F**). Altogether, these results suggest that lactate facilitates viral infection *via* suppressing plant defense responses.

## Discussion

In this study, we showed that SCMV utilizes the lactate fermentation pathway to promote its own infection. On the one hand, the LDH enzyme of the lactate fermentation pathway is recruited into VRCs by interacting with SCMV-encoded 6K2 protein. On the other hand, the accumulation of lactate, which is the end product of the lactate fermentation pathway, suppresses plant immunity to create favorable conditions for successful viral infection. Thus, this study reveals a viral pathogenic strategy by which to co-opt plant metabolism for not only acquiring replication components but also impairing host defense responses.

It is known that virus-triggered responses are associated with increasing demands for energy, reducing equivalents and carbon skeletons that are provided by host metabolic pathways, and are usually accompanied by the synthesis of defense substances (Lopez-Gresa et al., 2012; Fernandez-Calvino et al., 2014; Llave, 2016). In this study, differently expressed metabolites were mainly related to sulfur metabolism and riboflavin metabolism at the early stage of infection. Previously, hibiscus chlorotic ringspot virus-encoded CP can activate sulfur metabolism to trigger sulfur-enhanced resistance (Gao et al., 2012). Overexpressing riboflavin synthase in tobacco plants could increase resistance to tobacco mosaic virus (Zhao et al., 2014). Therefore, SCMV infection could alter the host defense-related metabolism at 4 dpi. Meanwhile, differently expressed metabolites at 5 dpi were mainly related to pyruvate metabolism, glycolysis and the TCA cycle, which may provide energy and building blocks for robust SCMV infection similarly to other viruses in previous reports (Plaxton, 1996; Nagy and Lin, 2020). In addition, several organic acids associated with the TCA cycle exhibit positive responses to plant viruses in different host species (Sidhu et al., 2010; Kogovsek et al., 2016). Therefore, maize metabolism is largely reconfigured for both defense responses and promoting viral multiplication during robust SCMV infection.

Pyruvate can be metabolized by anaerobic glycolysis and OxPhos after glucose metabolism. In this study, we found that SCMV infection can induce the activation of anaerobic glycolysis and the accumulation of lactate. Accordingly, we found that the accumulation levels of several intermediates of TCA cycle were decreased at 4 and 5 dpi, including fumaric acid, malate, alpha-ketoglutaric acid, succinic acid, as a result of the activation of anaerobic glycolysis (**Supplementary Figure 4**). In fact, some metabolites involved in OxPhs also play important roles during plant virus infection (Lopez-Gresa et al., 2012; Srivastava et al., 2012). Several studies have shown that intermediates of TCA cycle can be increased following plant virus infection, which could provide resources for host defense response or virus multiplication (Bazzini et al., 2011; Kogovsek et al., 2016). Recent study showed that lactate can be used as the fuel of TCA cycle in most mammalian tissues and cancer cells (Hui et al., 2017). Therefore, TCA cycle intermediates could be down-regulated by the activation of anaerobic glycolysis, meanwhile be up-regulated as lactate accumulates and the sugar metabolism activates during SCMV infection.

In this study, we found that the activation of anaerobic glycolysis before robust SCMV infection could promote SCMV infection. In contrast, the activation of oxidative phosphorylation before robust SCMV infection is not conducive to SCMV infection. The robust SCMV infection is dependent on rapid generation of ATP and production of new biomass in infected cells. The activation of anaerobic glycolysis allows for the rapid production of ATP locally by replenishing of the regulatory NAD^+^ pool in the end of glycolysis (Lin et al., 2019). Therefore, compared with oxidative phosphorylation, anaerobic glycolysis has faster ATP production efficiency, which benefits for SCMV replication. On the other hand, NAD^+^ and its reduced form NADH are also necessary for the biosynthesis of nucleotides and amino acids, and the rapid regeneration of NAD^+^ also allows fast incorporation of glucose metabolites into biomass (Vander Heiden et al., 2009; Lunt and Vander Heiden, 2011; Olson et al., 2016). Taken together, inducing anaerobic glycolysis rather than oxidative phosphorylation in pyruvate metabolism can support robust SCMV infection.

Virus infection causes profound metabolism changes in plants, which is thought to have a direct link to disease symptoms development (Pesti et al., 2019). In this study, our untargeted metabolomics analyses showed that, significantly changed metabolic pathways are mainly related to the synthesis of sulfur and riboflavin at 4 dpi, suggesting that the SCMV infection mainly affects some small molecular substances synthesis metabolism before the onset of symptoms. At 5 dpi, with the strong accumulation of SCMV and the appearance of symptoms, the number of differential metabolic pathways increase and most of which are mainly related to glycolysis and TCA. These data indicate that the symptoms appearance is closely related to the disorder of the respiratory metabolism. As we all known, mitochondrial electron transport chain (mETC) of respiratory metabolism is the primary source of ROS production (Andreyev et al., 2005). Previously, several studies showed that the mosaic and yellowing symptoms in virus-infected tissues are associated with ROS-induced peroxidation. For instance, the sharka symptom on plum pox virus-infected pea leaves is caused by a combination of a reduced antioxidant level and an increased ROS level (Diaz-Vivancos et al., 2006). The extent of oxidative stress and the antioxidant response in *N. benthamiana* plants positively correlate to the severity of the symptoms induced by pepper mild mottle virus (Hakmaoui et al., 2012). During bamboo mosaic virus infection, accumulation of H_2_O_2_ is restricted to symptomatic tissues in *N. benthamiana* and *Brachypodium distachyon* (Lin et al., 2021). In addition, the combined ROS generation from mitochondria has also been linked with programmed cell death (van Aken and van Breusegem, 2015; Huang et al., 2016; Zhao et al., 2018). Therefore, we conclude that the ROS burst caused by the disordered respiratory metabolism might play a key role in mosaic symptoms development.

In this study, overexpression or inhibition of LDH expression can accelerate or delay the time of symptoms appearance. Given that the appearance of symptoms is directly related to the disorder of plant metabolism (Llave, 2016), the contents of lactate may directly affect the time for symptoms appearance. Interestingly, several recent studies have highlighted the role of lactate as a fuel for the TCA cycle in cancer cells (Colegio et al., 2014; Hui et al., 2017). Therefore, in combination with the relationship between respiratory metabolism and symptoms discussed above, the reason why lactate affects the appearance of symptoms may be attributed to their function in fueling the TCA cycle, thereby affecting respiratory metabolism, further affecting ROS production, and leading to a change in the time for symptoms development.

Virus-hijacked cellular proteins are often used for developing and operating virus-driven structures, some of which provide a compartment for viral RNA replication (Nagy and Pogany, 2011; de Castro et al., 2013; Altan-Bonnet, 2017). During this process, virus must rewire cellular pathways to generate ATP for synthesizing molecular building blocks and RNA synthesis (Nagy and Lin, 2020). Glycolysis is a metabolic pathway that generates ATP in the cytoplasm, while its enzymes, including pyruvate kinase, glyceraldehyde-3-phosphate dehydrogenase, etc., can be recruited by tomato bushy stunt virus (TBSV) to the VRC for ATP production (Vander Heiden et al., 2010; Huang and Nagy, 2011; Chuang et al., 2017). Nevertheless, maintenance of glycolytic ATP production requires the replenishment of NAD^+^. A previous study found that TBSV P33 can replenish the regulatory NAD^+^ pool by interacting with PDC1 and ADH1 to co-opt the ethanol fermentation pathway (Lin et al., 2019). In this study, we discovered that the LDH2 enzyme, which is involved in the lactate fermentation pathway of glycolysis, colocalizes with SCMV-encoded 6K2 in VRCs. We propose that the role of LDH2 in SCMV VRCs should be similar as that of PDC1 and ADH1 in TBSV replication, i.e., to keep replenishing NAD^+^ so that ATP production can continue.In general, LDH expression is stimulated by either abiotic or biotic stresses, such as mechanical wounding, drought, cold stress, and *Botrytis cinerea* infection (Winter et al., 2007; Dolferus et al., 2008; Maurino and Engqvist, 2015). Here, we found that the enzymatic activity and transcriptional levels of ZmLDHs were significantly up-regulated in SCMV-infected plants at 4 and 5 dpi, which directly contributed to lactate accumulation. Given that virus could compartmentalize entire glycolytic/fermentation metabolism to promote intensive replication within the VRCs (Lin et al., 2019; Nagy and Lin, 2020), LDH could still catalyze pyruvate to produce lactate even if it is recruited into SCMV VRCs. Moreover, lactate plays important roles in the regulation of various cellular processes in animals (Colegio et al., 2014; Peng et al., 2016). In particular, lactate directly binds the mitochondrial antiviral signaling to prevent its aggregation and mitochondrial localization, thus to avoid innate immune recognition in mammalian cells during HBV infection (Zhou et al., 2021). In this study, lactate treatment suppressed the SCMV-induced plant defense responses by decreasing *PR* genes expression and H_2_O_2_ accumulation, which benefits SCMV infection. In fact, lactate can also enter and function in the mitochondria of plants (Paventi et al., 2007). Most recently, mitochondria are found as the main replication sites of SCMV (Xie et al., 2021). Considering that mitochondria function in multiple plant immunity pathways, including hormone-mediated immunity, programmed cell death, pathogen-associated molecular pattern-triggered immunity, effector-triggered immunity, and defense signal transduction, as well as the connection of mitochondria with other organelles in plant immunity (Wang et al., 2022), it will be interesting for further research to determine whether lactate has an impact on mitochondrion-mediated immune responses in plants.

## Data availability statement

The original contributions presented in the study are included in the article/**Supplementary Material**. Further inquiries can be directed to the corresponding authors.

## Author contributions

TJ and TZ designed the research. TJ conducted most of the experiments. KD, PW, and XW constructed vectors and cultured tobacco and maize plants. TJ, KD, LZ, DP, XC, GS, HZ, ZF, and TZ analyzed the data. TJ, ZC and TZ wrote the article. All the authors revised the article. All authors contributed to the article and approved the submitted version.

## References

[B1] Achon M. A. Serrano L. Alonso-Duenas N. Porta C. (2007). Complete genome sequences of maize dwarf mosaic and sugarcane mosaic virus isolates coinfecting maize in Spain. Arch. Virol. 152, 2073–2078. doi: 10.1007/s00705-007-1042-x 17680319

[B2] Akbar S. Yao W. Yu K. Qin L. Ruan M. Powell C. A.. (2020). Photosynthetic characterization and expression profiles of sugarcane infected by *Sugarcane mosaic virus* (SCMV). Photosynth. Res. 150, 279–294. doi: 10.1007/s11120-019-00706-w 31900791

[B3] Alegria O. M. Royer M. Bousalem M. Chatenet M. Peterschmitt M. Girard J. C.. (2003). Genetic diversity in the coat protein coding region of eighty-six sugarcane mosaic virus isolates from eight countries, particularly from Cameroon and Congo. Arch. Virol. 148, 357–372. doi: 10.1007/s00705-002-0916-1 12556998

[B4] Altan-Bonnet N. (2017). Lipid tales of viral replication and transmission. Trends Cell Biol. 27, 201–213. doi: 10.1016/j.tcb.2016.09.011 27838086 PMC5318230

[B5] Andreyev A. Y. Kushnareva Y. E. Starkov A. A. (2005). Mitochondrial metabolism of reactive oxygen species. Biochem. (Mosc) 70, 200–214. doi: 10.1007/s10541-005-0102-7 15807660

[B6] Bazzini A.A. Manacorda C.A. Tohge T. Conti G. Rodriguez M.C. Nunes-Nesi A.. (2011). Metabolic and miRNA profiling of TMV infected plants reveals biphasic temporal changes. PLoS One 6, e28466. doi: 10.1371/journal.pone.0028466 22174812 PMC3236191

[B7] Cao Y. Shi Y. Li Y. Cheng Y. Zhou T. Fan Z. (2012). Possible involvement of maize Rop1 in the defence responses of plants to viral infection. Mol. Plant Pathol. 13, 732–743. doi: 10.1111/j.1364-3703.2011.00782.x 22332840 PMC6638897

[B8] Chen H. Cao Y. Li Y. Xia Z. Xie J. Carr J. P.. (2017a). Identification of differentially regulated maize proteins conditioning *Sugarcane mosaic virus* systemic infection. New Phytol. 215, 1156–1172. doi: 10.1111/nph.14645 28627019

[B9] Cheng G. Dong M. Xu Q. Peng L. Yang Z. Wei T.. (2017). Dissecting the molecular mechanism of the subcellular localization and cell-to-cell movement of the *Sugarcane mosaic virus* P3N-PIPO. Sci. Rep. 7, 9868. doi: 10.1038/s41598-017-10497-6 28852157 PMC5575073

[B10] Chen L. Yan Z. Xia Z. Cheng Y. Jiao Z. Sun B.. (2017b). A violaxanthin deepoxidase interacts with a viral suppressor of RNA silencing to inhibit virus amplification. Plant Physiol. 175, 1774–1794. doi: 10.1104/pp.17.00638 29021224 PMC5717725

[B11] Chen H. Zou Y. Shang Y. Lin H. Wang Y. Cai R.. (2008). Firefly luciferase complementation imaging assay for protein-protein interactions in plants. Plant Physiol. 146, 368–376. doi: 10.1104/pp.107.111740 18065554 PMC2245818

[B12] Chuang C. K. Prasanth K. R. Nagy P. D. (2017). The glycolytic pyruvate kinase is recruited directly into the viral replicase complex to generate ATP for RNA synthesis. Cell Host Microbe 22, 639–652.e7. doi: 10.1016/j.chom.2017.10.004 29107644

[B13] Chung B. Y. Miller W. A. Atkins J. F. Firth A. E. (2008). An overlapping essential gene in the potyviridae. Proc. Natl. Acad. Sci. U.S.A. 105, 5897–5902. doi: 10.1073/pnas.0800468105 18408156 PMC2311343

[B14] Colegio O. R. Chu N. Q. Szabo A. L. Chu T. Rhebergen A. M. Jairam V.. (2014). Functional polarization of tumour-associated macrophages by tumour-derived lactic acid. Nature 513, 559–563. doi: 10.1038/nature13490 25043024 PMC4301845

[B15] Cotton S. Grangeon R. Thivierge K. Mathieu I. Ide C. Wei T.. (2009). Turnip mosaic virus RNA replication complex vesicles are mobile, align with microfilaments, and are each derived from a single viral genome. J. Virol. 83, 10460–10471. doi: 10.1128/JVI.00819-09 19656892 PMC2753101

[B16] de Castro I. F. Volonte L. Risco C. (2013). Virus factories: biogenesis and structural design. Cell. Microbiol. 15, 24–34. doi: 10.1111/cmi.12029 22978691 PMC7162364

[B17] Deng B. L. Jin X. H. Yang Y. Lin Z. W. Zhang Y. L. (2014). The regulatory role of riboflavin in the drought tolerance of tobacco plants depends on ROS production. Plant Growth Regul. 72, 269–277. doi: 10.1007/s10725-013-9858-8

[B18] Diaz-Vivancos P. Rubio M. Mesonero V. Periago P. M. Barcelo A. R. Martinez-Gomez P.. (2006). The apoplastic antioxidant system in *Prunus*: response to long-term plum pox virus infection. J. Exp. Bot. 57, 3813–3824. doi: 10.1093/jxb/erl138 17043083

[B19] Dolferus R. Ellis M. De Bruxelles G. Trevaskis B. Hoeren F. Dennis E. S.. (1997). Strategies of gene action in *Arabidopsis* during hypoxia. Ann. Bot. 79, 21–31. doi: 10.1093/oxfordjournals.aob.a010302

[B20] Dolferus R. Wolansky M. Carroll R. Miyashita Y. Ismond K. Good A. (2008). Functional analysis of lactate dehydrogenase during hypoxic stress in *Arabidopsis* . Funct. Plant Biol. 35, 131–140. doi: 10.1071/FP07228 32688764

[B21] Du K. Jiang T. Chen H. Murphy A. M. Carr J. P. Du Z.. (2020). Viral perturbation of alternative splicing of a host transcript benefits infection. Plant Physiol. 184, 1514–1531. doi: 10.1104/pp.20.00903 32958561 PMC7608148

[B22] Fan Z. F. Chen H. Y. Liang X. M. Li H. F. (2003). Complete sequence of the genomic RNA of the prevalent strain of a potyvirus infecting maize in China. Arch. Virol. 148, 773–782. doi: 10.1007/s00705-002-0964-6 12664299

[B23] Fernandez-Calvino L. Osorio S. Hernandez M. L. Hamada I. B. del Toro F. J. Donaire L.. (2014). Virus-induced alterations in primary metabolism modulate susceptibility to *Tobacco rattle virus* in arabidopsis. Plant Physiol. 166, 1821–U1991. doi: 10.1104/pp.114.250340 25358898 PMC4256867

[B24] Gao R. Ng F. K. Liu P. Wong S. M. (2012). *Hibiscus chlorotic ringspot virus* coat protein upregulates sulfur metabolism genes for enhanced pathogen defense. Mol. Plant Microbe Interact. 25, 1574–1583. doi: 10.1094/MPMI-08-12-0203-R 23134059

[B25] Hakmaoui A. Perez-Bueno M. L. Garcia-Fontana B. Camejo D. Jimenez A. Sevilla F.. (2012). Analysis of the antioxidant response of *Nicotiana benthamiana* to infection with two strains of *Pepper mild mottle virus* . J. Exp. Bot. 63, 5487–5496. doi: 10.1093/jxb/ers212 22915745 PMC3444274

[B26] Huang T. S. Nagy P. D. (2011). Direct inhibition of tombusvirus plus-strand RNA synthesis by a dominant negative mutant of a host metabolic enzyme, glyceraldehyde-3-phosphate dehydrogenase, in yeast and plants. J. Virol. 85, 9090–9102. doi: 10.1128/JVI.00666-11 21697488 PMC3165801

[B27] Huang S. Van Aken O. Schwarzlander M. Belt K. Millar A. H. (2016). The roles of mitochondrial reactive oxygen species in cellular signaling and stress response in plants. Plant Physiol. 171, 1551–1559. doi: 10.1104/pp.16.00166 27021189 PMC4936549

[B28] Hui S. Ghergurovich J. M. Morscher R. J. Jang C. Teng X. Lu W.. (2017). Glucose feeds the TCA cycle *via* circulating lactate. Nature 551, 115–118. doi: 10.1038/nature24057 29045397 PMC5898814

[B29] Hyodo K. Okuno T. (2016). Pathogenesis mediated by proviral host factors involved in translation and replication of plant positive-strand RNA viruses. Curr. Opin. Virol. 17, 11–18. doi: 10.1016/j.coviro.2015.11.004 26651023

[B30] Jiang J. X. Zhou X. P. (2002). Maize dwarf mosaic disease in different regions of China is caused by *Sugarcane mosaic virus* . Arch. Virol. 147, 2437–2443. doi: 10.1007/s00705-002-0890-7 12491109

[B31] Jiao Z. Y. Tian Y. Y. Wang J. Ismail R. G. Bondok A. Fan Z. F. (2022). Advances in research on maize lethal necrosis, a devastating viral disease. Phytopathol. Res. 4, 1–11. doi: 10.1186/s42483-022-00117-1

[B32] Kogovsek P. Pompe-Novak M. Petek M. Fragner L. Weckwerth W. Gruden K. (2016). Primary metabolism, phenylpropanoids and antioxidant pathways are regulated in potato as a response to *Potato virus y* infection. PloS One 11, e0146135. doi: 10.1371/journal.pone.0146135 26727123 PMC4738437

[B33] Li P. Guo L. Lang X. Li M. Wu G. Wu R.. (2022). Geminivirus C4 proteins inhibit GA signaling *via* prevention of NbGAI degradation, to promote viral infection and symptom development in *N. benthamiana* . PloS Pathog. 18, e1010217. doi: 10.1371/journal.ppat.1010217 35390110 PMC9060335

[B34] Lin W. Liu Y. Molho M. Zhang S. Wang L. Xie L.. (2019). Co-Opting the fermentation pathway for tombusvirus replication: Compartmentalization of cellular metabolic pathways for rapid ATP generation. PloS Pathog. 15, e1008092. doi: 10.1371/journal.ppat.1008092 31648290 PMC6830812

[B35] Lin K. Y. Wu S. Y. Hsu Y. H. Lin N. S. (2021). MiR398-regulated antioxidants contribute to *Bamboo mosaic virus* accumulation and symptom manifestation. Plant Physiol. 188, 593–607. doi: 10.1093/plphys/kiab451 PMC9040666 34695209

[B36] Livak K. J. Schmittgen T. D. (2001). Analysis of relative gene expression data using real-time quantitative PCR and the 2^-△△CT^ method. Methods 25, 402–408. doi: 10.1006/meth.2001.1262 11846609

[B37] Llave C. (2016). Dynamic cross-talk between host primary metabolism and viruses during infections in plants. Curr. Opin. Virol. 19, 50–55. doi: 10.1016/j.coviro.2016.06.013 27442236

[B38] Lopez-Gresa M. P. Lison P. Kim H. K. Choi Y. H. Verpoorte R. Rodrigo I.. (2012). Metabolic fingerprinting of tomato mosaic virus infected *Solanum lycopersicum* . J. Plant Physiol. 169, 1586–1596. doi: 10.1016/j.jplph.2012.05.021 22795749

[B39] Lunt S. Y. Vander Heiden M. G. (2011). Aerobic glycolysis: meeting the metabolic requirements of cell proliferation. Annu. Rev. Cell Dev. Biol. 27, 441–464. doi: 10.1146/annurev-cellbio-092910-154237 21985671

[B40] Mandal R. Kathiria P. Psychogios N. Bouatra S. Krishnamurthy R. Wishart D.. (2012). Progeny of tobacco mosaic virus-infected *Nicotiana tabacum* plants exhibit trans-generational changes in metabolic profiles. Biocatal. Agric. Biotechnol. 1, 115–123. doi: 10.1016/j.bcab.2012.01.004

[B41] Maurino V. G. Engqvist M. K. (2015). 2-hydroxy acids in plant metabolism. Arabidopsis Book 13, e0182. doi: 10.1199/tab.0182 26380567 PMC4568905

[B42] Meyer E. H. Welchen E. Carrie C. (2019). Assembly of the complexes of the oxidative phosphorylation system in land plant mitochondria. Annu. Rev. Plant Biol. 70, 23–50. doi: 10.1146/annurev-arplant-050718-100412 30822116

[B43] Mitsuya Y. Takahashi Y. Berberich T. Miyazaki A. Matsumura H. Takahashi H.. (2009). Spermine signaling plays a significant role in the defense response of *Arabidopsis thaliana* to cucumber mosaic virus. J. Plant Physiol. 166, 626–643. doi: 10.1016/j.jplph.2008.08.006 18922600

[B44] Nagy P. D. Lin W. (2020). Taking over cellular energy-metabolism for TBSV replication: the high ATP requirement of an RNA virus within the viral replication organelle. Viruses 12, 56. doi: 10.3390/v12010056 31947719 PMC7019945

[B45] Nagy P. D. Pogany J. (2011). The dependence of viral RNA replication on co-opted host factors. Nat. Rev. Microbiol. 10, 137–149. doi: 10.1038/nrmicro2692 22183253 PMC7097227

[B46] Olson K. A. Schell J. C. Rutter J. (2016). Pyruvate and metabolic flexibility: Illuminating a path toward selective cancer therapies. Trends Biochem. Sci. 41, 219–230. doi: 10.1016/j.tibs.2016.01.002 26873641 PMC4783264

[B47] Pallas V. Garcia J. A. (2011). How do plant viruses induce disease? interactions and interference with host components. J. Gen. Virol. 92, 2691–2705. doi: 10.1099/vir.0.034603-0 21900418

[B48] Paventi G. Pizzuto R. Chieppa G. Passarella S. (2007). L-lactate metabolism in potato tuber mitochondria. FEBS J. 274, 1459–1469. doi: 10.1111/j.1742-4658.2007.05687.x 17489101

[B49] Peng M. Yin N. Chhangawala S. Xu K. Leslie C. S. Li M. O. (2016). Aerobic glycolysis promotes T helper 1 cell differentiation through an epigenetic mechanism. Science 354, 481–484. doi: 10.1126/science.aaf6284 27708054 PMC5539971

[B50] Pesti R. Kontra L. Paul K. Vass I. Csorba T. Havelda Z.. (2019). Differential gene expression and physiological changes during acute or persistent plant virus interactions may contribute to viral symptom differences. PloS One 14, e0216618. doi: 10.1371/journal.pone.0216618 31051010 PMC6499435

[B51] Plaxton W. C. (1996). The organization and regulation of plant glycolysis. Annu. Rev. Plant Physiol. Plant Mol. Biol. 47, 185–214. doi: 10.1146/annurev.arplant.47.1.185 15012287

[B52] Rosenwasser S. Mausz M. A. Schatz D. Sheyn U. Malitsky S. Aharoni A.. (2014). Rewiring host lipid metabolism by large viruses determines the fate of *Emiliania huxleyi*, a bloom-forming alga in the ocean. Plant Cell 26, 2689–2707. doi: 10.1105/tpc.114.125641 24920329 PMC4114960

[B53] Sade D. Shriki O. Cuadros-Inostroza A. Tohge T. Semel Y. Haviv Y.. (2014). Comparative metabolomics and transcriptomics of plant response to *Tomato yellow leaf curl virus* infection in resistant and susceptible tomato cultivars. Metabolomics 11, 81–97. doi: 10.1007/s11306-014-0670-x

[B54] Sagor G. H. Takahashi H. Niitsu M. Takahashi Y. Berberich T. Kusano T. (2012). Exogenous thermospermine has an activity to induce a subset of the defense genes and restrict cucumber mosaic virus multiplication in *Arabidopsis thaliana* . Plant Cell Rep. 31, 1227–1232. doi: 10.1007/s00299-012-1243-y 22371256

[B55] Sharma M. Sasvari Z. Nagy P. D. (2010). Inhibition of sterol biosynthesis reduces tombusvirus replication in yeast and plants. J. Virol. 84, 2270–2281. doi: 10.1128/JVI.02003-09 20015981 PMC2820916

[B56] Sidhu O. P. Annarao S. Pathre U. Snehi S. K. Raj S. K. Roy R.. (2010). Metabolic and histopathological alterations of *Jatropha mosaic begomovirus*-infected *Jatropha curcas* l. by HR-MAS NMR spectroscopy and magnetic resonance imaging. Planta 232, 85–93. doi: 10.1007/s00425-010-1159-0 20372923

[B57] Srivastava S. Bisht H. Sidhu O. P. Srivastava A. Singh P. C. Pandey R. M.. (2012). Changes in the metabolome and histopathology of *Amaranthus hypochondriacus* l. @ in response to *Ageratum enation virus* infection. Phytochemistry 80, 8–16. doi: 10.1016/j.phytochem.2012.05.007 22683210

[B58] van Aken O. van Breusegem F. (2015). Licensed to kill: mitochondria, chloroplasts, and cell death. Trends Plant Sci. 20, 754–766. doi: 10.1016/j.tplants.2015.08.002 26442680

[B59] Vander Heiden M. G. Locasale J. W. Swanson K. D. Sharfi H. Heffron G. J. Amador-Noguez D.. (2010). Evidence for an alternative glycolytic pathway in rapidly proliferating cells. Science 329, 1492–1499. doi: 10.1126/science.1188015 20847263 PMC3030121

[B60] Vander Heiden M.G. Cantley L.C. Thompson C.B. (2009). Understanding the Warburg effect: the metabolic requirements of cell proliferation. Science 324, 1029–1033. doi: 10.1126/science.1160809 19460998 PMC2849637

[B61] Wang A. (2015). Dissecting the molecular network of virus-plant interactions: the complex roles of host factors. Annu. Rev. Phytopathol. 53, 45–66. doi: 10.1146/annurev-phyto-080614-120001 25938276

[B62] Wang J. Xu G. Ning Y. Wang X. Wang G. L. (2022). Mitochondrial functions in plant immunity. Trends Plant Sci. 27, 1063–1076. doi: 10.1016/j.tplants.2022.04.007 35659746

[B63] Wang R. Yang X. Wang N. Liu X. Nelson R. S. Li W.. (2016). An efficient virus-induced gene silencing vector for maize functional genomics research. Plant J. 86, 102–115. doi: 10.1111/tpj.13142 26921244

[B64] Wei T. Huang T. S. McNeil J. Laliberte J. F. Hong J. Nelson R. S.. (2010). Sequential recruitment of the endoplasmic reticulum and chloroplasts for plant potyvirus replication. J. Virol. 84, 799–809. doi: 10.1128/JVI.01824-09 19906931 PMC2798358

[B65] Winter D. Vinegar B. Nahal H. Ammar R. Wilson G. V. Provart N. J. (2007). An "electronic fluorescent pictograph" browser for exploring and analyzing large-scale biological data sets. PloS One 2, e718. doi: 10.1371/journal.pone.0000718 17684564 PMC1934936

[B66] Wyrsch I. Dominguez-Ferreras A. Geldner N. Boller T. (2015). Tissue-specific FLAGELLIN-SENSING 2 (FLS2) expression in roots restores immune responses in arabidopsis *fls2* mutants. New Phytol. 206, 774–784. doi: 10.1111/nph.13280 25627577

[B67] Xia Z. Zhao Z. Chen L. Li M. Zhou T. Deng C.. (2016). Synergistic infection of two viruses MCMV and SCMV increases the accumulations of both MCMV and MCMV-derived siRNAs in maize. Sci. Rep. 6, 20520. doi: 10.1038/srep20520 26864602 PMC4808907

[B68] Xie J. Jiang T. Li Z. Li X. Fan Z. Zhou T. (2021). Sugarcane mosaic virus remodels multiple intracellular organelles to form genomic RNA replication sites. Arch. Virol. 166, 1921–1930. doi: 10.1007/s00705-021-05077-z 33905022

[B69] Yang M. Ismayil A. Jiang Z. Wang Y. Zheng X. Yan L.. (2022). A viral protein disrupts vacuolar acidification to facilitate virus infection in plants. EMBO J. 41, e108713. doi: 10.15252/embj.2021108713 34888888 PMC8762549

[B70] Yuan W. Jiang T. Du K. Chen H. Cao Y. Xie J.. (2019). Maize phenylalanine ammonia-lyases contribute to resistance to *Sugarcane mosaic virus* infection, most likely through positive regulation of salicylic acid accumulation. Mol. Plant Pathol. 20, 1365–1378. doi: 10.1111/mpp.12817 31487111 PMC6792131

[B71] Zhao Y. Luo L. Xu J. Xin P. Guo H. Wu J.. (2018). Malate transported from chloroplast to mitochondrion triggers production of ROS and PCD in *Arabidopsis thaliana* . Cell Res. 28, 448–461. doi: 10.1038/s41422-018-0024-8 29540758 PMC5939044

[B72] Zhao Y. Y. Wang D. F. Wu T. Q. Guo A. Dong H. S. Zhang C. L. (2014). Transgenic expression of a rice riboflavin synthase gene in tobacco enhances plant growth and resistance to tobacco mosaic virus. Can. J. Plant Pathol. 36, 100–109. doi: 10.1080/07060661.2014.881921

[B73] Zhou L. He R. Fang P. Li M. Yu H. Wang Q.. (2021). Hepatitis b virus rigs the cellular metabolome to avoid innate immune recognition. Nat. Commun. 12, 1–13. doi: 10.1038/s41467-020-20316-8 33397935 PMC7782485

[B74] Zhu M. Chen Y. Ding X. S. Webb S. L. Zhou T. Nelson R. S.. (2014). Maize elongin c interacts with the viral genome-linked protein, VPg, of *Sugarcane mosaic virus* and facilitates virus infection. New Phytol. 203, 1291–1304. doi: 10.1111/nph.12890 24954157 PMC4143955

