# Supplementary material for: Sugarcane mosaic virus orchestrates the lactate fermentation pathway to support its successful infection
Source: Front Plant Sci. 2023 Jan 9;13:1099362. doi: 10.3389/fpls.2022.1099362 (PMC9868461; doi:10.3389/fpls.2022.1099362)
Supplement: Supplementary file 1 [file DataSheet_1.pdf]

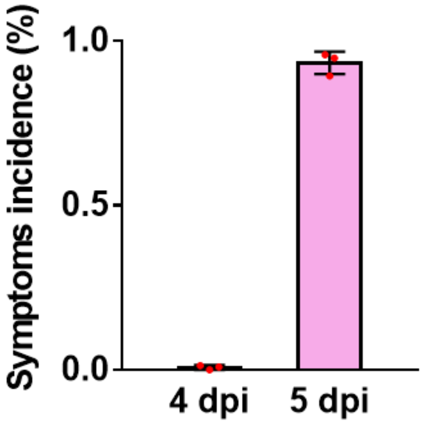

Supplementary Figure 1. Statistical analysis of the numbers of plants showing mosaic symptoms at 4 and 5 dpi. Most inoculated plants showed mosaic symptoms on the first systemically-infected leaf at 5 dpi.

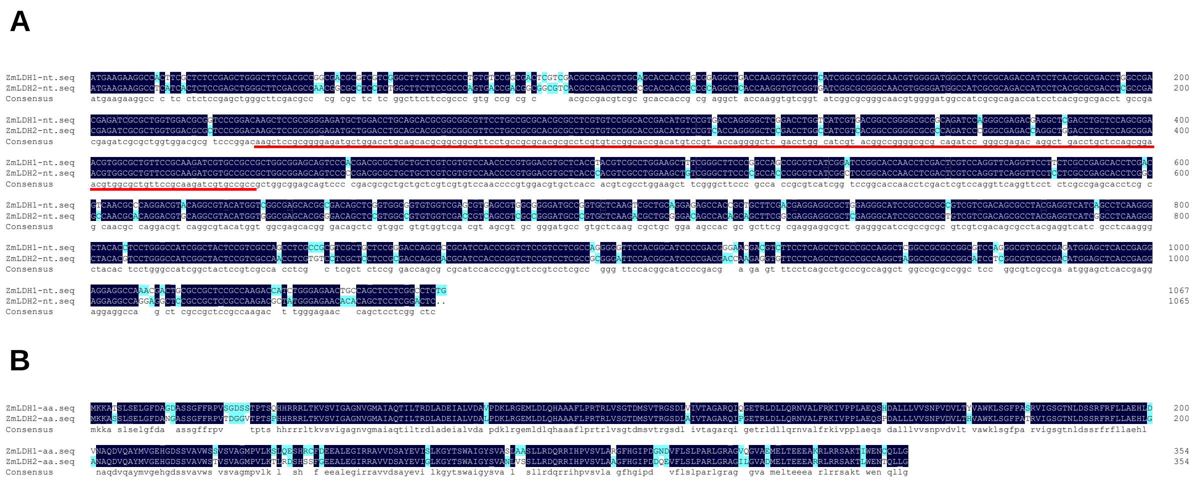

Supplementary Figure 2. Multiple nucleotide and amino acid sequences alignment of ZmLDH. **(A)** Multiple nucleotide sequences alignment showed high identity between *ZmLDH1* and *ZmLDH2*. The selected fragment for VIGS was underlined.

(B) Multiple amino acid sequences alignment showed high identity between ZmLDH1 and ZmLDH2.

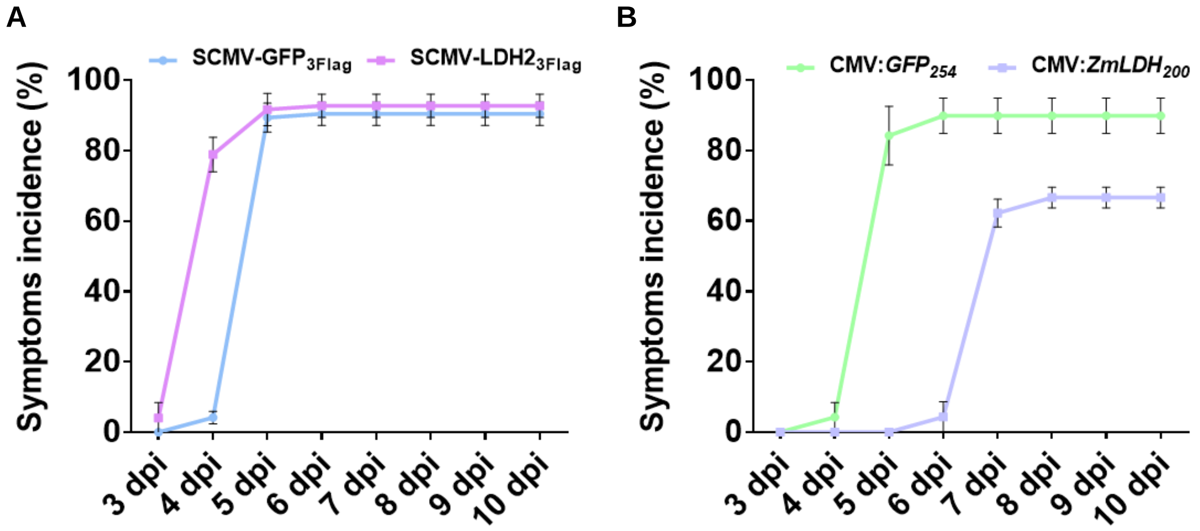

Supplementary Figure 3. Incidences of plants with mosaic symptoms at different time points post inoculation.

(A) Statistical analysis of the numbers of SCMV-GFP_{3Flag} or SCMV-$LDH2_{3Flag}$-infected plants showing mosaic symptoms.

(B) Statistical analysis of the numbers of GFP or *ZmLDH*-silenced plants showing mosaic symptoms.

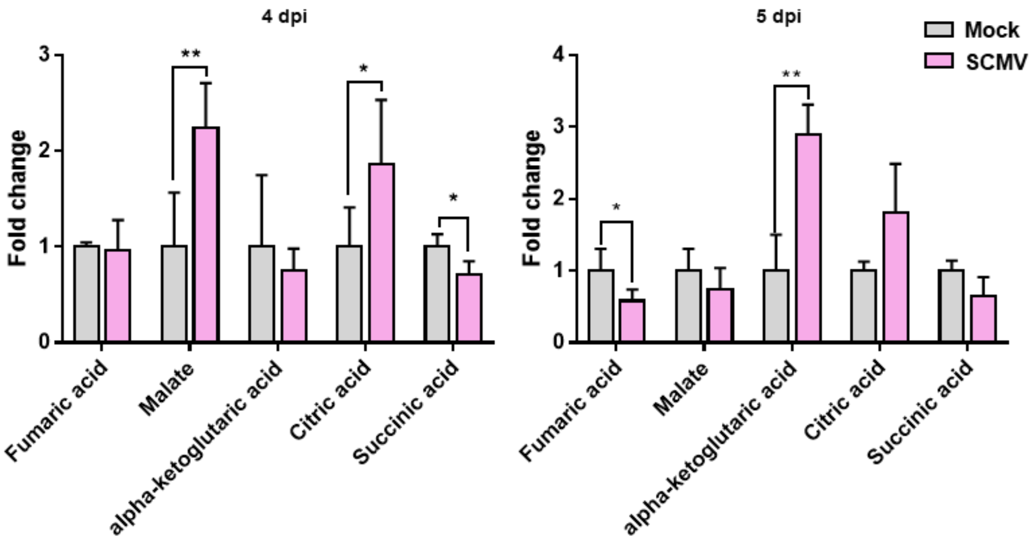

Supplementary Figure 4. Fold change of TCA metabolite levels in SCMV-infected plants over mock-inoculated plants at 4 and 5 dpi.

Untargeted metabolomics was used to analyze changes in TCA intermediates. The data are represented as the means ± SE (n=4). Statistical differences between the treatments were determined using unpaired Student's *t*-test (two-tailed), *, $P < 0.05$; **, $P < 0.01$.
